# Gut microbiota and OMVs: Unveiling novel regulatory mechanisms in high‐altitude myocardial injury

**DOI:** 10.1002/imo2.70001

**Published:** 2025-02-05

**Authors:** Mingyang Chang, Yongqiang Zhou, Tiantian Xia, Pan Shen, Ningning Wang, Chaoji Huangfu, Zhijie Bai, Dezhi Sun, Yangyi Hu, Shuman Li, Zhexin Ni, Wei Zhou, Yue Gao

**Affiliations:** ^1^ Department of Pharmaceutical Sciences Beijing Institute of Radiation Medicine Beijing China; ^2^ Tianjin Key Laboratory of Translational Research of TCM Prescription and Syndrome First Teaching Hospital of Tianjin University of Traditional Chinese Medicine Tianjin China; ^3^ Medical College of Qinghai University Xining China; ^4^ State Key Laboratory of Kidney Diseases, Chinese PLA General Hospital Beijing China

## Abstract

The gut microbiota influences host health, with outer membrane vesicles (OMVs) facilitating intercellular communication and transporting bioactive molecules. At high altitudes, OMVs can cross the intestinal barrier, affecting heart function and potentially modulating inflammation, oxidative stress, and apoptosis in cardiomyocytes. Genetic engineering of OMVs may improve their therapeutic efficacy by altering surface properties to enhance targeting and residence time. They could also act as disease biomarker carriers for early detection and monitoring.

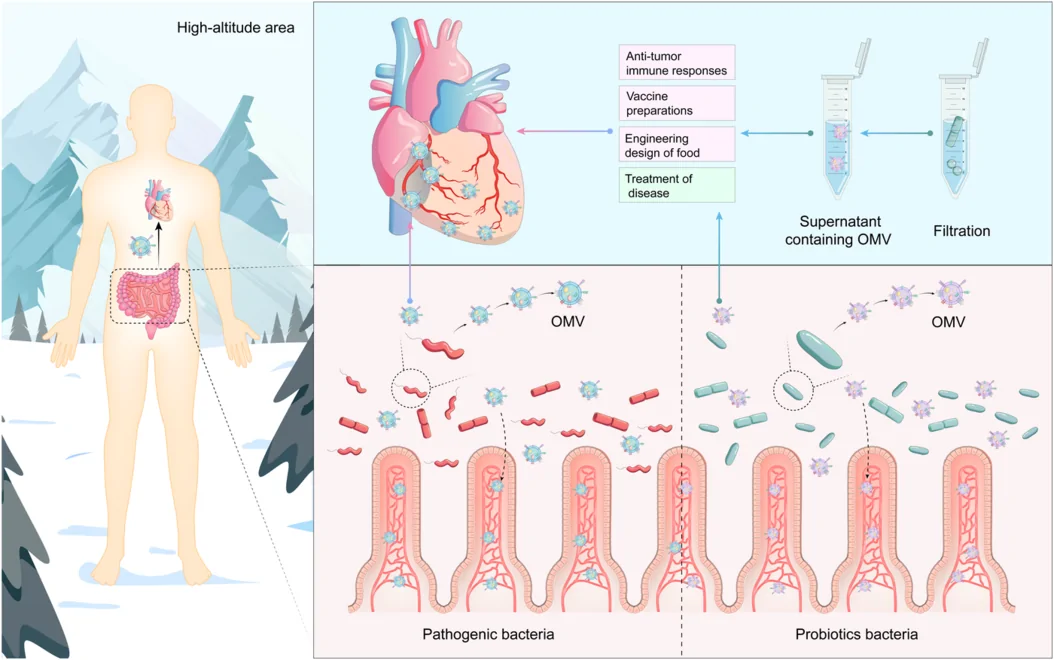

## CONFLICT OF INTEREST STATEMENT

The authors declare no conflicts of interest.

## 1 ETHICS STATEMENT

No animals or humans were involved in this study.

To the Editor,

In the context of high‐altitude environments, characterized by hypoxia, low temperature, and reduced atmospheric pressure, the gut microbiota undergoes substantial changes that can adversely impact human health. This paper posits that outer membrane vesicles (OMVs), originating from the gut microbiota, may act as a critical conduit for the interaction between the microbiota and host physiology, and could be a pivotal area for future biotechnological innovation.

## 1 THE GUT MICROBIOTA PLAYS A VITAL ROLE IN REGULATING HUMAN HEALTH

The gut microbiota, which refers to the collective microbiome present within the human gastrointestinal tract, encompasses viruses, bacteria, fungi, and other microscopic organisms. This community is comprised of up to 100 trillion bacterial cells, which is an order of magnitude greater than the number of human cells. The gut microbiota is home to between 400 and 500 distinct species, with these microorganisms engaging in complex interactions with the human body [1]. The gut microbiota plays a crucial role in maintaining human health, involving various organs such as the intestines, liver, pancreas, brain, and a range of diseases including obesity, cancer, and diabetes [2]. Previous studies have indicated alterations in gut function among patients with chronic heart failure, suggesting that the gut microbiota exerts an influence on myocardial cells [3].

## 2 PLATEAU ENVIRONMENT LEADS TO SIGNIFICANT CHANGES IN GUT MICROBIOTA

Hypobaric hypoxia is the principal environmental challenge faced by populations in plateau regions, significantly impacting the physiological functions of the inhabitants. The gut microbiota undergoes changes when confronted with this hypoxic environment, alterations that can have a substantial impact on the host's susceptibility to diseases [4]. As insights into the interactions between gut microbiota and the host advance, researchers are investigating ways to modulate the gut microbiota to adapt to and alleviate the disease burden associated with hypoxia. Our research group is dedicated to investigating the impact of high‐altitude environments and gut microbiota on disease. The latest published article from our team reveals that under hypoxic conditions, alterations in the gut microbiota are causally linked to cognitive dysfunction, highlighting the association between gut microbiota and brain‐related disorders [5]. An unpublished manuscript from our group, utilizing multi‐omics analysis, has identified associations between the gut microbiota and its metabolites with high‐altitude heart disease, indicating that under hypoxic conditions, the gut microbiota influences cardiac health.

## 3 MYOCARDIAL INJURY IN HIGH‐ALTITUDE ENVIRONMENTS: A COMMON AND CRITICAL HEALTH CHALLENGE

**Figure 1:**
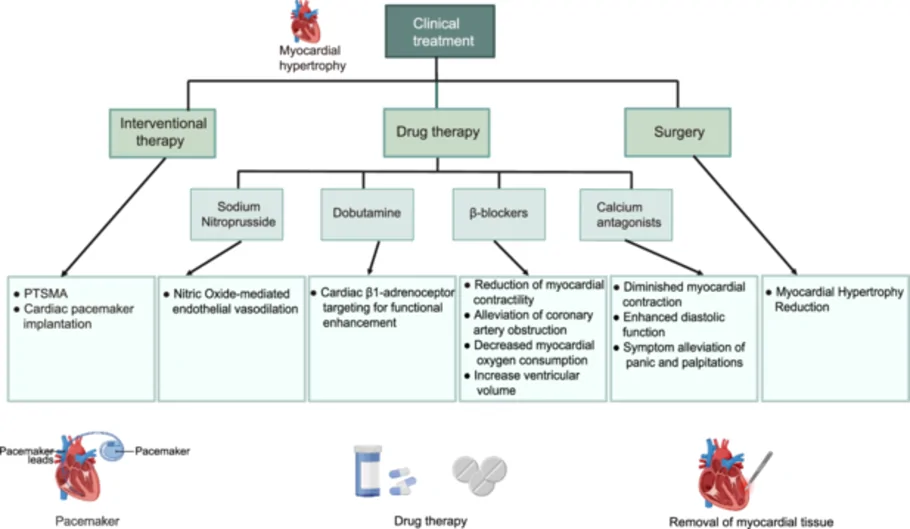
**Treatment approaches for high‐altitude myocardial injury in clinical practice.** The treatment approaches for HMI include interventional therapy, drug therapy, and surgery. Interventional therapy: This encompasses cardiac pacemaker implantation and percutaneous transluminal septal myocardial ablation (PTSMA). Drug therapy: The main drugs used are as follows: Sodium Nitroprusside: A vasodilator that stimulates the endothelium to release nitric oxide. Dobutamine: A drug that targets the β1‐adrenergic receptors in the heart. β‐blockers: These reduce myocardial contraction, alleviate coronary artery obstruction, decrease myocardial oxygen consumption, and increase ventricular volume. Calcium antagonists: These reduce myocardial contraction and improve diastolic function. Surgery: This includes removing the myocardium.

With the development of society and economy, an increasing number of individuals are relocating to high‐altitude areas for work and study. However, the sustained high‐altitude environment can induce hypoxic damage to vital organs, including the heart and brain [6, 7]. The heart plays a crucial role in blood circulation, delivering oxygen‐rich blood to all tissues and organs. Prolonged exposure to hypoxic conditions often leads to cardiac hypertrophy and cardiac dysfunction. Myocardial injury is characterized by the impairment or necrosis of cardiomyocytes, manifesting as elevated levels of cardiac troponin I or T exceeding the 99th percentile upper reference limit [8]. A variety of factors can lead to the occurrence of myocardial injury, and in the clinical setting, there are numerous therapeutic approaches to myocardial injury, as depicted in Figure 1.

## 4 OMVS: THE SURPRISING LINK BETWEEN GUT MICROBIOTA DISRUPTION AND HEART HEALTH

**Figure 2:**
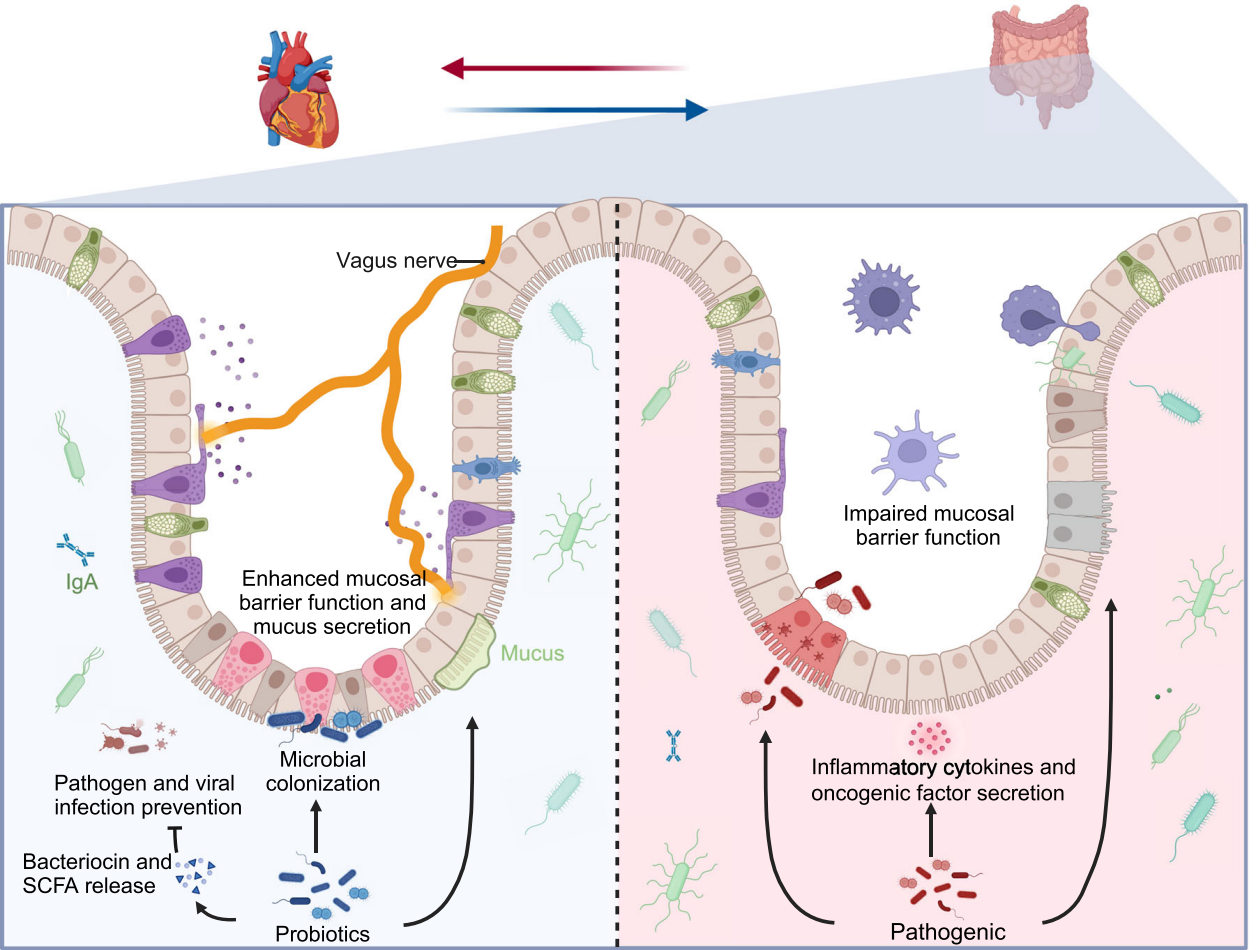
**Role of gut microbiota and outer membrane vesicles in high‐altitude.** The gut microbiota plays a crucial role in host health, with OMVs facilitating intercellular communication and bioactive molecule transport. At elevated altitudes, OMVs can penetrate the intestinal barrier, influencing heart function and potentially modulating inflammation, oxidative stress, and apoptosis in cardiomyocytes. Modifying OMVs through genetic engineering may enhance their therapeutic potential by altering surface properties to improve targeting and prolong residence time in the body. OMVs have the potential to serve as carriers for disease biomarkers, enabling early detection and disease monitoring.

Despite the existence of various factors leading to myocardial injury, the pathogenic mechanisms and therapeutic approaches under hypoxic conditions remain relatively limited. Building upon previous research, our team has identified the gut microbiota as a potential therapeutic target for high‐altitude myocardial injury (HMI). Upon focusing on this aspect, we have further discovered that the OMVs secreted by the gut microbiota play a significant role in the pathogenesis of HMI. Consequently, our research group is currently investigating this area to explore novel pathogenic mechanisms of HMI and to develop new therapeutic strategies. We anticipate uncovering the underlying molecular pathogenic mechanisms and providing novel approaches for targeted disease prevention and control, as depicted in Figure 2.

## 5 OMVS OF THE GUT MICROBIOTA AS MEDIATORS OF MICROBIAL COMMUNICATION IN HMI

Research on bacterium‐host communication has primarily focused on mechanisms involving cell surface attachment and internalization, where bioactive molecules are directly delivered from bacteria to host cells via various secretion systems [9]. Numerous Gram‐negative bacteria release OMVs both in vitro and in vivo [10, 11, 12]. These nanometer‐scale particles are significant contributors to bacterial pathogenicity. For now, there are over a thousand narratives on OMVs. In recent years, research on OMVs has shown an upward trend. Initially, OMVs were considered products of cell lysis, a notion that persisted until 1975, when their presence was observed in the cerebrospinal fluid samples of patients with acute meningitis. Since that discovery, OMVs have garnered widespread attention in the scientific community [13]. The canonical pathway for the biogenesis of OMVs involves blebbing of the outer membrane of Gram‐negative bacteria. Hence, OMVs are enriched with numerous outer membrane proteins and specific lipid components [14]. These constituents provide the foundation for their dissemination within the body, enabling them to travel away from the intestines and reach target organs. However, the presence of nucleic acids (such as DNA and RNA) and enzymes within OMVs has not yet been fully substantiated. Due to their small size and membranous structure, which facilitate their presence within the body, OMVs can serve as intercellular communication molecules, participating in the delivery of toxins, transfer of DNA, and transportation of bacterial antigenic materials [15]. Thus, as a tool, OMVs hold significant potential in modulating the progression of various diseases. However, due to the current limitations in research, a specific pathway by which OMVs regulate the onset of diseases has not yet been identified. This highlights the need for further investigation to elucidate the precise mechanisms through which OMVs can influence disease processes. Such research could pave the way for the development of targeted therapeutic applications utilizing OMVs in the future.

## 6 UNDERSTANDING AND HARNESSING THE FORMATION OF OMVS

Currently, four mechanisms have been identified for the derivation of OMVs in Gram‐negative bacteria. The first mechanism involves the cross‐linking of peptidoglycan (PG) and the outer membrane. The Gram‐negative bacterial cell wall comprises an outer membrane, an inner membrane, and a periplasmic lumen situated between the two membranes. A thin layer of PG connects the inner and outer membranes. When bacteria encounter specific stimuli, lipoprotein (LPP) undergoes denaturation and hydrolysis, resulting in alterations to the PG structure. This change decreases the cross‐linking bonds between PG and LPP, which subsequently triggers the formation of OMVs. The second mechanism is related to periplasmic accumulation. Misplaced periplasmic proteins can exert pressure on the outer membrane, leading to the formation of OMVs. The third mechanism, known as bilayer coupling, involves the deacylation of lipid A at the outer membrane. This process causes membrane remodeling and increases the curvature of the outer membrane, facilitating OMVs formation. The final mechanism is gene‐related. When the *VacJ* and/or *yrb* genes are silenced or absent, phospholipids (PL) accumulate in the outer leaflets of the outer membrane. This accumulation results in asymmetric expansion of the outer leaflets, promoting the outgrowth of the outer membrane to form OMVs. OMVs are spherical bilayered proteolipid structures secreted by Gram‐negative bacteria, which have attracted increasing attention in the fields of microbiology, immunology, and biotechnology due to their diverse biological functions and potential applications. A comprehensive understanding of the mechanisms underlying OMVs production is crucial for harnessing their therapeutic and intervention potential. With a clearer definition of the mechanisms involved in OMVs production, researchers can leverage this knowledge to enhance targeted OMVs production for therapeutic or intervention purposes (Figure S1).

## 7 EXPLORING OMVS OF THE GUT MICROBIOTA: FROM PATHOGENIC MECHANISMS TO THERAPEUTIC INNOVATIONS

Given the diversity and complexity of bacterial envelopes, the mechanisms involved in biogenesis are not yet fully understood. Understanding the commonalities and specific characteristics of OMVs from different bacteria will aid in the development of OMVs. Recent studies suggest that OMVs have the potential to be engineered into vaccines that stimulate the innate immune system for disease prevention and control [16, 17, 18] (Figure S2). However, their application in the context of heart diseases is still largely unexplored. Hence, determining if OMVs can target the heart, understanding their precise mechanisms of action, and leveraging their properties for the development of novel pharmaceuticals are pressing issues that demand persistent research efforts. These questions also form the central theme of our team's forthcoming investigative work.

To summarize, at high altitudes, the gut microbiota plays a crucial role in the pathophysiology of high‐altitude myocardial injury. This condition is characterized by a decline in cardiac performance, increased inflammation, and the death of myocardial cells. these microorganisms release OMVs that are capable of traversing the intestinal barrier and modulating heart function through interactions with pathways implicated in inflammation, oxidative stress, and apoptosis. The investigation of the mechanisms by which OMVs influence cardiac health is an important and rapidly evolving area of research. Understanding these interactions could provide valuable insights into the development of novel therapeutic strategies for managing high‐altitude myocardial injury and improving cardiovascular outcomes in such environments.

## AUTHOR CONTRIBUTIONS

**Mingyang Chang**: Writing–review and editing; writing–original draft; validation; investigation; visualization. **Yongqiang Zhou**: Writing–original draft; validation; visualization. **Tiantian Xia**: Visualization; validation; writing–original draft. **Pan Shen**: Visualization; investigation. **Ningning Wang**: Visualization; investigation. **Chaoji Huangfu**: Investigation; visualization. **Zhijie Bai**: Visualization; investigation. **Dezhi Sun**: Investigation; visualization. **Yangyi Hu**: Investigation; visualization. **Shuman Li**: Investigation; visualization. **Zhexin Ni**: Investigation; writing–review and editing; validation; supervision. **Wei Zhou**: Writing–review and editing; investigation; validation; supervision. **Yue Gao**: Writing–review and editing; funding acquisition; validation; supervision; investigation.

## Supporting information

The online version contains supplementary figures and tables available.

**Figure S1:** Mechanisms of outer membrane vesicles (OMVs) formation in gram‐negative bacteria.
**Figure S2:** Extraction, separation and function of outer membrane vesicles (OMVs).

## Data Availability

Data sharing not applicable to this article as no datasets were generated or analysed during the current study. No new data were generated in this study. Supplementary materials (figures, graphical abstract, slides, videos, Chinese translated version and update materials) may be found in the online DOI or iMetaOmics http://www.imeta.science/imetaomics/.
