# Supplementary material for: Gut microbiota and OMVs: Unveiling novel regulatory mechanisms in high‐altitude myocardial injury
Source: IMetaOmics. 2025 Feb 5;2(1):e70001. doi: 10.1002/imo2.70001 (PMC12806435; doi:10.1002/imo2.70001)
Supplement: Supplementary file 1 — Figure S1: Mechanisms of outer membrane vesicles (OMVs) formation in gram‐negative bacteria. Figure S2: Extraction, separation and function of outer membrane vesicles (OMVs). [file IMO2-2-e70001-s001.docx]

**Supporting information to:**

**Gut Microbiota and OMVs: Unveiling Novel Regulatory Mechanisms in High-Altitude Myocardial Injury**

**Running title:** Gut Microbiota and OMVs in High-Altitude Myocardial Injury

Mingyang Chang^1, 2#^, Yongqiang Zhou^1#^, Tiantian Xia^3#^, Pan Shen^1^, Ningning Wang^1^, Chaoji Huangfu^1^, Zhijie Bai^1^, Dezhi Sun^1^, Yangyi Hu^1^, Shuman Li^1^, Zhexin Ni^1*^, Wei Zhou^1*^, Yue Gao^1, 4*^

^1^Department of Pharmaceutical Sciences, Beijing Institute of Radiation Medicine, Beijing, 100080, China

^2^Tianjin Key Laboratory of Translational Research of TCM Prescription and Syndrome, First Teaching Hospital of Tianjin University of Traditional Chinese Medicine, Tianjin 300193, China

^3^Medical College of Qinghai University, Xining 810016, China

^4^State Key Laboratory of Kidney Diseases, Chinese PLA General Hospital, Beijing, 100080, China

^#^These authors contributed equally: Mingyang Chang, Yongqiang Zhou, Tiantian Xia

^*^Correspondence: gaoyue@bmi.ac.cn (Yue Gao), zhouweisyl802@163.com (Wei Zhou) and [nizxzg@163.com](mailto:nizxzg@163.com) (Zhexin Ni)

**Supplementary figures:**

**Figure S1 Mechanisms of outer membrane vesicles (OMVs) formation in gram-negative bacteria.** (1) peptidoglycan (PG) cross-linking with the outer membrane is disrupted by lipoprotein (LPP) denaturation and hydrolysis, reducing PG-LPP bonds and triggering OMVs formation. (2) Periplasmic protein accumulation exerts pressure on the outer membrane, inducing OMVs formation. (3) Deacylation of lipid A in the outer membrane causes membrane remodeling and increased curvature, promoting OMVs formation.

**Figure S2 Extraction, separation and function of outer membrane vesicles (OMVs).** OMVs are commonly derived from bacterial supernatants using methods like ultrafiltration coupled with centrifugation, immunoaffinity chromatography, and detergent-based extraction for attenuated OMVs (dOMVs). The characterization of OMVs involves the use of various techniques such as WB SDS-PAGE, ELISA, AFM, SEM, TEM, NTA, DLS. The functions of OMVs can be harnessed for genetic engineering, drug delivery, and vaccine development. WB: Western Blot, SDS-PAGE: Sodium Dodecyl Sulfate-Polyacrylamide Gel Electrophoresis, ELISA: Enzyme-Linked Immunosorbent Assay, AFM: Atomic Force Microscopy, SEM: Scanning Electron Microscopy, TEM: Transmission Electron Microscopy, NTA: Nanoparticle Tracking Analysis, DLS: Dynamic Light Scattering.
